# AXL and CAV-1 play a role for MTH1 inhibitor TH1579 sensitivity in cutaneous malignant melanoma

**DOI:** 10.1038/s41418-019-0488-1

**Published:** 2020-01-09

**Authors:** Ishani Das, Helge Gad, Lars Bräutigam, Linda Pudelko, Rainer Tuominen, Veronica Höiom, Ingrid Almlöf, Varshni Rajagopal, Johan Hansson, Thomas Helleday, Suzanne Egyházi Brage, Ulrika Warpman Berglund

**Affiliations:** 1 Department of Oncology–Pathology, Karolinska Institutet, S-171 64 Stockholm, Sweden; 2 Department of Oncology–Pathology, Science for Life Laboratory, Karolinska Institutet, S-171 64 Stockholm, Sweden; 3 Department of Oncology, Karolinska University Hospital, S-171 76 Stockholm, Sweden; 4 Department of Oncology and Metabolism, Weston Park Cancer Centre, University of Sheffield, Sheffield, S10 2RX UK

**Keywords:** Oncogenes, Tumour biomarkers

## Abstract

Cutaneous malignant melanoma (CMM) is the deadliest form of skin cancer and clinically challenging due to its propensity to develop therapy resistance. Reactive oxygen species (ROS) can induce DNA damage and play a significant role in CMM. MTH1 protein protects from ROS damage and is often overexpressed in different cancer types including CMM. Herein, we report that MTH1 inhibitor TH1579 induced ROS levels, increased DNA damage responses, caused mitotic arrest and suppressed CMM proliferation leading to cell death both in vitro and in an in vivo xenograft CMM zebrafish disease model. TH1579 was more potent in abrogating cell proliferation and inducing cell death in a heterogeneous co-culture setting when compared with CMM standard treatments, vemurafenib or trametinib, showing its broad anticancer activity. Silencing MTH1 alone exhibited similar cytotoxic effects with concomitant induction of mitotic arrest and ROS induction culminating in cell death in most CMM cell lines tested, further emphasizing the importance of MTH1 in CMM cells. Furthermore, overexpression of receptor tyrosine kinase AXL, previously demonstrated to contribute to BRAF inhibitor resistance, sensitized *BRAF* mutant and *BRAF/NRAS* wildtype CMM cells to TH1579. AXL overexpression culminated in increased ROS levels in CMM cells. Moreover, silencing of a protein that has shown opposing effects on cell proliferation, CAV-1, decreased sensitivity to TH1579 in a BRAF inhibitor resistant cell line. AXL-MTH1 and CAV-1-MTH1 mRNA expressions were correlated as seen in CMM clinical samples. Finally, TH1579 in combination with BRAF inhibitor exhibited a more potent cell killing effect in *BRAF* mutant cells both in vitro and in vivo. In summary, we show that TH1579-mediated efficacy is independent of *BRAF/NRAS* mutational status but dependent on the expression of AXL and CAV-1.

## Introduction

Cutaneous malignant melanoma (CMM) is responsible for most number of skin cancer related deaths [1]. Since the discovery of the role of MAPK signaling pathway in melanoma genesis, several BRAF, and MEK inhibitors (BRAFi and MEKi) have been approved and used to treat the ~50% of patients who have CMM harboring *BRAFV600* mutations. Treatment efficacy to MAPK pathway targeting therapy of advanced *BRAF*-mutated CMM is high, but often not long-lasting due to resistance development [2]. A more comprehensive understanding of the immune system in recent years has led to the development of checkpoint inhibitors and has in many cases become first line treatment with long-term effects observed for only a subset of the patients [3, 4]. Hence there is still an unmet need for finding alternative treatment options.

Recent work suggests that CMM to some extent is a Reactive oxygen species (ROS) driven tumor as CMM cells exhibit high levels of ROS and ROS-induced DNA damage that may cause genetic alterations [5]. Accumulating evidence illustrates a link between MAPK signaling pathway, ROS, and DNA damage responses [7]. BRAFi treatment has been shown to induce ROS [6], indicating that ROS may play a role in the pharmacological response of BRAFi and in the development of resistance to the treatment [7]. Thus, ROS can promote cancer development and cancer cell survival [8–10], but interestingly also suppress tumor growth through induction of apoptosis and senescence via DNA damage [11].

DNA repair is a safeguard for protecting cells against the deleterious effects of ROS. Tumor cells often upregulate several DNA repair proteins, one of them being MTH1, to maintain the genomic integrity. MTH1 sanitizes the oxidized dNTP pool by hydrolyzing harmful oxidative stress induced 8-oxo-dGTP, thus preventing its incorporation into DNA [12]. MTH1 has been shown to be upregulated in a number of cancer types including CMM [13–15]. Although tumor cells have elevated levels of MTH1, normal cells are less dependent on it [16]. We and others [16, 17] hypothesized that the upregulated level of MTH1 is a salvage pathway for the cancer cell to survive and escape the elevated ROS-induced oxidative damage and apoptosis/senescence. However, the pharmacology and biology of MTH1 and its inhibitors seems to be more complex than we originally reported, since other groups developed noncytotoxic MTH1 inhibitors [18], while also additional cytotoxic MTH1 inhibitors have been reported [19]. Further work is needed to help refine our understanding in this area. Wang et al. [15] demonstrated that silencing MTH1 made *BRAF* mutant CMM cells more susceptible to oxidative stress induced apoptosis.

Resistance to BRAFi has been associated with reactivation of the MAPK pathway stemming from upregulation of RTKs such as AXL [20–23], which has been associated with resistance to DNA damaging therapies [24]. The scaffolding protein caveolin-1 (CAV-1) has also been associated to drug resistance [25] and to integrate transduction of multiple signaling including MAPK cascade [26].

In this study we investigated the cytotoxic potential of TH1579 in CMM cells. Using FACS and time lapse we were able to show induction of cell death and mitotic arrest upon treatment with TH1579. AXL and CAV-1 played a role in mediating TH1579 sensitivity. AXL-CAV-1 and MTH1 are correlated, which was further validated in a CMM patient cohort. Lastly, we show that combining BRAFi with TH1579 was more effective in killing *BRAF* mutant CMM cells. Our study highlights novel mechanisms underlying TH1579-mediated cytotoxicity.

## Material and methods

### Clinical samples

Tumors from 32 CMM patients have previously been sampled (fresh frozen core or fine needle aspirates) prior to onset of treatment with MAPK targeting therapy or checkpoint inhibitors and from five of the patients a sample was collected during treatment from the same tumor. Twenty of the patients were male and twelve female. Median age of the patients was 66 years (range 42–86 years). The CMM were classified as stage IV M1a (*n* = 3), M1b (*n* = 5), and M1c (*n* = 24). This study has been approved by the regional ethics committee in Stockholm, Sweden and has been conducted in accordance with the ethical principles given in the Helsinki Declaration. Informed consent was obtained from all the patients.

### Cell lines and reagents

#### Cell culture

A375PR1 and A375VR4 were BRAFi resistant cell lines derived from A375 American Type Culture Collection (ATCC), where A375PR1 was induced with PLX4720 and A375VR4 was induced with vemurafenib [27]. *NRAS* mutant SkMel2 (Q61R) was obtained from ATCC, whereas ESTDAB102 (Q61R), ESTDAB149 (Q61R), and *BRAF/NRAS* wildtype (WT) cell lines ESTDAB105, ESTDAB138 were obtained from European Searchable Tumor Line Database and Cell Bank (ESTDAB). For all experiments, CMM patient-derived cell lines 159-PRE (pretreatment short-term patient-derived cell line generated in house originating from fine needle aspirates) were cultured in DMEM. *BRAF* mutant cell lines were cultured in MEM supplemented while the *NRAS* and *BRAF/NRAS* WT cell lines were cultured in RPMI-1640. For co-cultures, spheroids, shMTH1 lines, cell lines generated with histone H2B tags and in vivo transplants all cells were cultured in DMEM. All cell lines were cultured as per the manufacturer’s guidelines (Thermo scientific) and confirmed to be mycoplasma free using LookOut Mycoplasma PCR detection kit (Sigma-Aldrich, Stockholm, Sweden).

### Florescent labeling of cells

Using lentiviral transfection with pLenti-CMV-blast plasmids, A375 and SkMel2 cells were transfected with eGFP, A375VR4, and ESTDAB102 cells with mTagBFP and ESTDAB105 cells with mKO2. Stable cells were generated by antibiotic selection with 4 µg/mL blasticidin for 7 days.

### H2B cells

A375 and A375VR4 cells were transfected by lentivirus using the H2b-GFP pLenti-CMV hygro plasmid. Stable cells were generated by antibiotic selection with 400 µg/mL hygromycin for 7 days.

### Plasmids, cell lines, siRNA, and shRNA

All CMM cells used for in vivo zebrafish injections were stably expressing tdTomato and luciferase or eGFP, and all shMTH1 cell lines were generated by a lentivirus-based approach using the vector #32904, #17477 (Addgene) for in vivo injections and Ginseng vector [28] for shMTH1 cells. To select cells successfully transfected with the shRNA vector, puromycin at 4 µg/mL was used for 7 days. To induce the shRNA expression, cells were treated with doxycycline at 1 µg/mL for 5–6 days. All siRNA and overexpression plasmids were transfected using Lipofectamine 2000 (Sigma-Aldrich Chemie Gmbh, Munich, Germany) according to the manufacturer’s recommendations. pBluescript PMS2 was a gift from Bert Vogelstein (Addgene #16457), pX1 was a gift from Monica Hollstein (Addgene #46848), pIRES-EGFP- puro was a gift from Michael McVoy (Addgene #45567) and pIRES –puro2 AXL was gift from Aaron Meyer (Addgene #65627). H2b-GFP was subcloned by PCR into the pENTR1A-GFP-N2 plasmid (Addgene #19364) at the HindII and BamH1 restriction sites. The H2b-GFP pENTR1A plasmid was verified by sequencing and H2b-GFP was shuttled into the pLenti-CMV hygro DEST plasmid (Addgene #17454) by LR clonase. mTagBFP2-pENTR1A vector were generated by subcloning the mTagBFP2 ORF into the pENTR1A no ccDB (Addgene #17398) vector by PCR using the XhoI and XbaI restriction sites. pLENTI-CMV-blast vectors with either mTagBFP2, eGFP or mKO2 were generated by shuttling the mTagBFP2-pENTR1A, pENTR1A-GFP-N2, and mKO2-N1 vectors into the pLenti-CMV-blast DEST vector using LR clonase. pENTR1A-GFP-N2 (FR1), pENTRIA no ccDB (w48-1), and pLenti-CMV Hygro DEST (w117-1) were gifts from Eric Campeau and Paul Kaufman. mTagBFP2-pBAD was a gift from Michael Davidson (Addgene plasmid #54572). mKO2-N1 was from Michael Davidson and Atsushi Miyawaki (Addgene plasmid #54625).

### In vivo transplantation, drug treatment, and luciferase measurement

All cell cultures were harvested 1 h prior to transplantation into zebrafish embryos. For a detailed description on preparation of cells cultures, please view respective reference [29]. Directly before transplantation, a highly concentrated cell suspension was loaded into nonfilament microcapillaries (World Precision Instruments) and ~100 cells were injected into the blastula of zebrafish embryos at 2 h post fertilization. Transplanted embryos were transferred into E3 medium in a 10 cm culture dish and incubated at 33 °C. The next day, embryos were screened for successful transplantation, dechorionized using Pronase (Sigma) and distributed into six-well plates (25 embryos/well) in a total volume of 3 mL E3 medium containing 25 mM HEPES. MTH1 inhibitor TH1579 (dissolved in DMSO to 10 mM) was added directly to the medium to a final concentration of 20 or 40 μM. DMSO was used as control. For combination experiments, the embryos were divided into four groups (DMSO control, Vemurafenib (10 µM), TH1579 (20 µM) and combination). During treatment, embryos were incubated at 33 °C until individual tumor size detection by luminescence measurement. After drug exposure, single zebrafish embryos were transferred into opaque 96-well plates (Perkin Elmer) and incubated for 30 min in lysis buffer (10% glycerol, 1% Triton X-100, 1 mM DTT, pH 7.8). An equal amount of substrate solution (1 mM DTT, 1 mM ATP, 0.3 mg/mL d-luciferin, pH 7.8) was added for 5 min before measurement of luminescence (Hidex Sense). For every animal experiment conducted, 5–10 embryos were used per group to ensure statistical power. For the combination studies, only animals that had tumors above the median value for that group were included in the analysis. For all other studies, all animals were included. Zebrafish embryos were randomized into different treatment groups at the start of the treatment. No blinding was performed.

### Flow cytometry

Fluorescence signal for Annexin V and PI-staining (Sigma-Aldrich Chemie Gmbh, Munich, Germany), or Andy Fluor 647 Annexin V (BioCat Gmbh Heidelberg, Germany) for comparing sets between co-culture and single culture experiments using fluorescently labeled cells was measured by flow cytometry (Novocyte 3000). A minimum of 10,000 events were measured using polygonal gating to exclude debris. Fluorescence intensity was analyzed using Novoexpress software (ACEA Biosciences, San Diego, CA, USA) to determine induction of apoptosis and necrosis.

### Cell cycle analysis

For cell cycle analysis, 100,000 cells/well were plated overnight in 12-well plates, treated with either DMSO or 0.9 µM TH1579 for 24 h. The cells were then trypsinized, harvested, and fixed in 4% buffered formaldehyde for 18 h at room temperature, followed by fixation in 95% ethanol for 1 h, and, finally, rehydrated in distilled water for 1 h. After treatment with subtilisin Carlsberg solution (0.1% Sigma protease XXIV. 0.1 M Tris, and 0.07 M NaCl (pH 7.2)) and staining with DAPI-Sulforhodamine solution (8 µM DAPI, 50 µM sulforhodamine 101, 0.1 M Tris, and 0.07 M NaCl (pH 7.5)), samples were analyzed using a LSRII flow cytometer (Becton Dickinson), equipped with a UV laser. DAPI fluorescence was measured above 435 nm. Cell nuclei sub-G1 DNA content served as indicators of apoptotic cell death. The ModFit program for cell cycle analysis (Verity software house) was used for histogram analysis. The number of nuclei/histogram was 10,000.

### 2D proliferation assay

For calculating the synergy index scores for the drug combination experiments, 800–1000 cells/well were plated overnight in a 384-well plate and DMSO as control or drugs were dispensed using a D300 digital dispenser (Hewlett-Packard, Tecan Trading AG, Switzerland). After 72 h treatment of the cells, resazurin (Sigma-Aldrich Chemie Gmbh, Munich, Germany) was added and relative fluorescence was measured using a plate reader (Tecan Spark 10 M, Tecan Trading AG, Switzerland) at 530–570 nm (excitation), and at 590–620 nm (emission). Synergy scores were calculated using Synergy Finder web application (https://synergyfinder.fimm.fi).

### 2D MTS assay

A total of 3000–4000 cells/well was plated overnight in 96-well flat bottomed plates. Next day, cells were exposed to either TH1579 for 72 h after which MTS solution (Promega, Madison, WI, USA) was added and absorbance at 490 nm was measured using Tecan Spark 10 M plate reader (Tecan Trading AG, Switzerland) to determine the inhibitory concentration of the drugs according to the manufacturer’s protocol.

### 3D MTS assay using tumor sphere growth with the hanging drop method

Approximately 10,000 cells/well were pipetted into conical well ULA plates (Corning art. 7007, Sigma-Aldrich Chemie Gmbh, Munich, Germany) in DMEM medium. To each well with 200 µl media and cells, additional medium was added to overfill the wells. Lids were attached using spacers to allow room for the hanging drops before turning the plates. Plates were shaken at 300 rpm with amplitude of 3 mm on a lab shaker (Thermo Scientific, Waltham, MA, USA) at 37 °C in cell incubator overnight. Plates were turned back, excess media removed, and the spheres were left to mature for 3–5 days, before being treated with single drug for 72 h. 3D MTS solution CellTiter 3D (Promega, Madison, WI, USA) was added according to the manufacturer’s protocol. The plate was wrapped in aluminum foil and mixed at 30 rpm on a laboratory rocker (Thermo Fisher Scientific, Waltham, MA, USA) for cell lysis (30 min at 37 °C). Fifty microliters of the lysate were read on a luminescence plate for ATP determination in Tecan Spark 10 M microplate reader (Tecan Trading AG, Switzerland) and drug efficacy on viability. CDI values were calculated as for the 2D viability assays.

### Colony formation assay

A total of 1000 cells/well were plated into six-well plates overnight. Cells were treated with either DMSO or 0.9 µM TH1579 for 5 days, with media change after every 2 days. Colonies were allowed to form for an additional 7 days in absence of the drug, with the media being replaced every 3 days. Cells were after 12 days fixed for 20 min using 4 % buffered formaldehyde. Colonies were stained with 0.05% crystal violet solution for 10 min followed by two washes with 1X PBS. Stained plates were scanned using Epson scanner V370. For estimation of the number of colonies formed, crystal violet was dissolved in 100% methanol, transferred to a 96-well plate, diluted 1:10 using PBS and absorbance was measured at 540 nm using Tecan Spark 10 M plate reader instrument.

### ROS measurement

A total of 50,000 cells/well were incubated overnight in 12-well plates. For ROS measurements, cells were either left untreated or were treated with TH1579 (0.5 or 0.9 µM) or, PLX4032 (0.45 µM) or the combination, trypsinzed and stained with CM-H2DCF (Life Technologies, C8627) and analyzed by FACS as per the manufacturer’s protocol.

### Modified comet assay

The modified comet assay was performed as earlier described [16]. Briefly, cells were seeded into six-well plates at a density of 150,000–200,000 cells per well and the day after treated for 24 h. Cells were then harvested by trypsinization and washed with 1X PBS, resuspended in 1X PBS at a concentration of approximately one million cells/ml. Cell suspension was mixed with 1.2 % low melting agarose and the mixture was added over to 1% agarose coated fully frosted slides. The slides were incubated in lysis buffer containing [100 mM sodium EDTA, 2.5 mM NaCl, 10 mM Tris–HCl (pH 10)], 1% Triton X-100 and 10% DMSO 2 h at room temp in the dark. After incubation, slides were washed three times with enzyme buffer containing KCl, Hepes, EDTA, and BSA followed by addition of OGG1 enzyme and incubated at 37 °C for 45 min. At the end of incubation period alkaline denaturation with alkali buffer (300 mM NaOH, 1 mM sodium EDTA) was carried out in an electrophoresis chamber for 20 min. Electrophoresis was run at 25 V and 300 mA in the same buffer for 30 min. The slides were later neutralized with neutralizing buffer [250 mM Tris–HCl (pH 7.5)] for 45 min. Just before imaging, the slides were stained with 1X SYBR gold dye. One hundred comets were account using Comet IV software.

### Immunoblotting

For western blots, cells were lysed on ice using RIPA buffer (25 mM Tris•HCl pH 7.6, 150 mM NaCl, 1% NP-40, 1% sodium deoxycholate, 0.1% SDS), 1 mM NaOV, protease and phosphatase inhibitors for 30 min and vortexed every 10 min followed by centrifugation and protein measurement using BCA kit as per the manufacturer’s protocol. Protein was loaded on NuPage 4–12% Bis-Tris gel (Thermo Fisher Scientific, Waltham, MA, USA) and transferred into a PVDF membrane. Membranes were blocked in 5% BSA and incubated overnight with primary antibodies followed by incubation with secondary antibodies detected by ECL reagent using Image Quant LAS 4000 (GE Healthcare Europe GmbH, Freiburg, Germany).

### Immunofluorescence

Cryosections using zebrafish embryos were made as previously described [30]. For immunostaining on cryosections, the slides were equilibrated in 1X PBS-Tween 20 for three washes, 5 min each, and then blocked with 10% donkey serum (ab 7475) or goat serum (ab 7481) in 1X PBS-Tween 20 for 1 h at room temperature. After blocking, the slides were incubated overnight at 4 °C with either anti-CAV-1 (1:400) or anti-AXL (1:100). Next day, the slides were washed in 1X PBS-Tween 20 for three washes, 5 min each with minimal shaking (50 rpm). They were incubated with secondary antibody (1:500) for 2 h followed by 1X PBS-Tween 20 washes for three times, 5 min each. The slides were then mounted using DAPI fluoroshield (F6057) and imaged by AxioImager M2 (Zeiss).

### Time-lapse microscopy

A total of 700–1200 cells/well either single or in co-culture were plated in a 96-well black plate with transparent bottom, treated with DMSO or PLX4032 (0.4 µM) or Trametinib (3 nM) or TH1579 (0.45, 0.9, 1.8 µM) and incubated at 37 °C in 95% humidified atmosphere with 5% CO_2_. The plates were imaged at 10X using time-lapse microscopy (ImageXpress MicroXL (Molecular Devises)) between days 0 and 4 at a regular interval of 24 h. Images were then exported and quantified using cell profiler analyst software. The cell numbers were calculated based on staining the cells with DAPI. All cell numbers for each group were normalized to the cell count for that group on day 0.

### RNA extraction

Cell line RNA extraction was performed using the product manual using the AllPrep DNA/RNA/miRNA kit (Qiagen, Hilden, Germany). RNA quantity and quality measurements were performed using Agilent Bioanalyzer 2100 instrument (Agilent Technologies Inc., Santa Clara, CA, USA).

### Real-time PCR

The extracted RNA was converted to cDNA in a 20 µl reaction using standard reagents from Invitrogen with SuperScript III reverse transcriptase (Carlsbad, CA, USA). The cDNA was diluted fourfold with ddH2O and subjected to semiquantitative real-time PCR reaction in a Bio-Rad CFX instrument (Hercules, CA, USA). The real-time PCR results were analyzed in CFX Manager software.

### Extraction of data from targeted sequencing using Ion AmpliSeq™

Targeted sequencing of fine needle aspirate or core biopsy RNA from metastases and RNA from cell lines was previously performed using the Ion AmpliSeq Transcriptome Human Gene Expression Kit for RefSeq genes ((Thermo Fisher Scientific, Waltham, MA. USA) as described in [27]. Data on mRNA abundance of our candidates of interest were extracted from the normalized transcript using the reads per kilobase per million reads method.

### Co-immunoprecipitation assay

Cells were plated at 85% confluency and allowed to grow overnight. The following day, the cells were collected and protein was extracted on ice using cell lysis buffer (cell signaling, #9803) with protease and phosphatase supplements and protein amount was quantified using BCA as previously described. A total of 300 µg of protein was mixed with prewashed magnetic beads (Thermo Scentific, #88802) and primary antibody (concentration used as per the manufacturer’s recommendation) and incubated overnight at 4 °C. The following day, beads were washed again to remove unbound antibody, boiled with 4X SDS (Thermo Scientific, #NP0007) and analyzed using western blot.

### In vitro kinase assay

A total of 384-well, white, low volume, nonbinding plates (Costar #3824) were nanodispensed with dose–response curves of test compounds. The AXL kinase reaction (V3961, Promega) was performed using 1× reaction buffer, containing 50 µM DTT, including AXL kinase (1.2 µg/mL), AXLtide substrate (0.2 mg/mL), and ATP (50 µM) in a total volume of 5 µl/well and a reaction time of 60 min at room temperature followed by the ADP-Glow assay (V9101, Promega) where 5 µl/well of ADP-Glo reagent was added and plate was incubated at room temperature for 40 min after which 10 µl/well of kinase detection reagent was added followed by 30 min incubation at room temperature. Luminescence was recorded in Hidex Sense reader.

### CETSA-western blot

CETSA (Cellular Thermal Shift Assay) was performed as previously described in [31]. Briefly, cells were plated overnight to 70% confluency. The following day, cells were treated with DMSO or TH1579 (0.9 µM) for 2 h at 37 °C followed by trypsinization and resuspension in media. A total of 80 µl of cells were added to a PCR tube per heating condition and heat treated for 3 min (Veriti 96-well thermal cycler, AB). Samples were then lysed using RIPA buffer with required supplements. Samples were stored at −80 °C, until further analysis by western blot.

### *In situ* PLA

*In situ* PLA was run using Duolink In situ PLA Sigma-Aldrich (according to the manufacturer’s protocol). Briefly, the cells were permeabilized in 0.5% Triton X-100 in TBS for 5 min followed by washing in TBST (TBS + 0.05% Tween 20). Blocking was performed overnight. Antibody (primary) incubation was performed O/N. After PLA the cells were stained with 1:40 Phalloidin for 15 min at room temperature and mounted with mounting medium containing DAPI. Cells were then analyzed using florescence microscope (Zeiss) and subsequently the PLA signal was measured using cell profiler software.

Lot Numbers:

Antibody diluent: SLBT2003/SLBX5745.

Blocking solution: SLBV6065.

Anti rabbit minus probe: Cat No DUO82005 lot nr SLBX3145.

Anti rabbit plus probe: Cat No DUO82002 lot nr SLBT8716.

Anti mouse plus probe: Cat no DUO82001 lot no SLBV2113.

Anti mouse minus probe Cat no DUO82004 lot no SLBS7468.

### Statistical analysis

All experiments were performed in triplicate and representative results were presented where data were expressed as mean ± SD or mean ± SEM as mentioned in figure legends. Variance between groups statistically compared was similar. All statistical analyses were carried out using GraphPad Prism v.7.0 (GraphPad Software, La Jolla, CA, USA). Two-tailed student’s *t* test or two-way ANNOVA test was used to compare the difference between groups. For comparing patient survival data, Mantel–Cox log rank test was employed. For all other comparisons between patient groups, Mann–Whitney *U* test was used.

## Results

### Sensitivity to TH1579 is independent of *BRAF/NRAS* mutational background

**Fig. 1:**
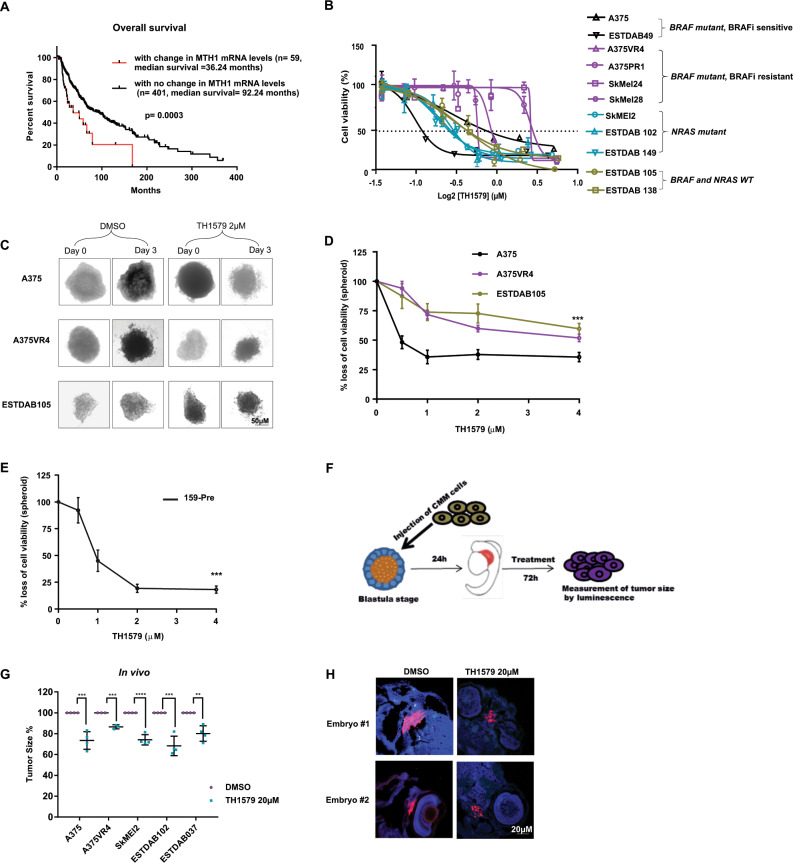
CMM cells are sensitive to MTH1 inhibitor TH1579 independent of *BRAF/NRAS* mutational status. **a** High MTH1 mRNA expression significantly decreases overall survival in CMM patients (*n* = 460) (data analyzed from TCGA). **b** Cell viability measured by MTS following 72 h treatment with TH1579 shows that *NRAS, BRAF* mutated and WT CMM are sensitive to TH1579 treatment. CMM cells with acquired or intrinsic resistance to vemurafenib have higher IC50 values for TH1579. (Error bars represent mean ± SEM; *n* = 3). **c** Representative images taken before (day 0) and after 72 h (day 3) of treatment showing the effects of 2 µM TH1579 on CMM spheroid compared with control (DMSO) treated spheroids. **d** After 72 h treatment with 0, 0.5, 1, 2 or 4 µM TH1579, the cell viability of the spheroids were measured by CellTiterGlo. (Error bars represent mean ± SD; *n* = 3, ***p < 0.001, two-way ANNOVA test). **e** Short-term patient-derived cell line, 159-Pre (*BRAF* WT) was cultured as spheroids and treated with TH1579 at concentrations shown in the figure. After 72 h treatment, cell viability was measured by CellTiterGlo. (Error bars represent mean ± SD; *n* = 3, ***p < 0.001, two-way ANNOVA test). **f** Schematic illustration of the experimental model of CMM  transplanted zebrafish embryo disease model used in this study. Briefly, ~100 CMM cells stably expressing tdTomato and luciferase were injected into blastula of zebrafish embryo. The next day, embryos were screened for successful transplantation, and distributed into six-well plates (15 embryos/well). TH1579 was added directly to the medium to a final concentration of 20 or 40 μM. After 72 h, individual embryos were lysed and amount of CMM cells (i.e., tumor volume) were measured by luminescence. **g** TH1579 (20 μM) significantly reduces tumor volume in CMM transplanted zebrafish embryo disease model. Tumor size calculated as % of reduction of DMSO control. Data shown as mean ± SD from *n* = 4 independent experiments (Error bars represent mean ± SD, **p < 0.01, ***p < 0.001, ****p < 0.0001, Student’s *t* test). **h** Loss of Ki67 signal shown by IF of zebrafish embryo sections transplanted with SkMel2 collected after 72 h treatment with TH1579 (20 µM).

Given that ROS and oxidative stress has been suggested to drive CMM development and MTH1 sanitizes oxidized nucleotides, we wanted to see if MTH1 levels correlated with prognosis of CMM. Analysis of the TCGA dataset revealed that CMM patients with higher mRNA levels of MTH1 (greater than twofold) exhibit a shorter disease-free survival (*p* = 0.053) (Supplementary Fig. S1a) and a significantly shorter overall survival (*p* < 0.001) (Fig. 1a), suggesting a role of MTH1 as a prognostic marker for CMM.

To investigate whether the *BRAF/NRAS* mutational background of CMM influences the response to TH1579, CMM cells were exposed for 72 h and cell viability was measured. The inhibitory concentration at 50% (IC50) showed a variation from 0.23 to 1.4 µM (Supplementary Table S1a). BRAFi resistant cell lines had the highest IC50 values when compared with the remaining cell lines (Fig. 1b and Supplementary Table S1).

The cytotoxic effects of TH1579 were confirmed in a spheroid model system (Fig. 1c, d). A primary patient-derived cell line 159-Pre (*BRAF* WT) also responded very well to TH1579 treatment (Fig. 1e). TH1579 significantly reduced tumor growth as well as the levels of Ki67 in a zebrafish model (*p* < 0.01) without showing general toxicity at the concentration tested (Fig. 1f–h and Supplementary Fig. S1b, c).

### Heterogenous CMM co-cultures are sensitive to TH1579 treatment

**Fig. 2:**
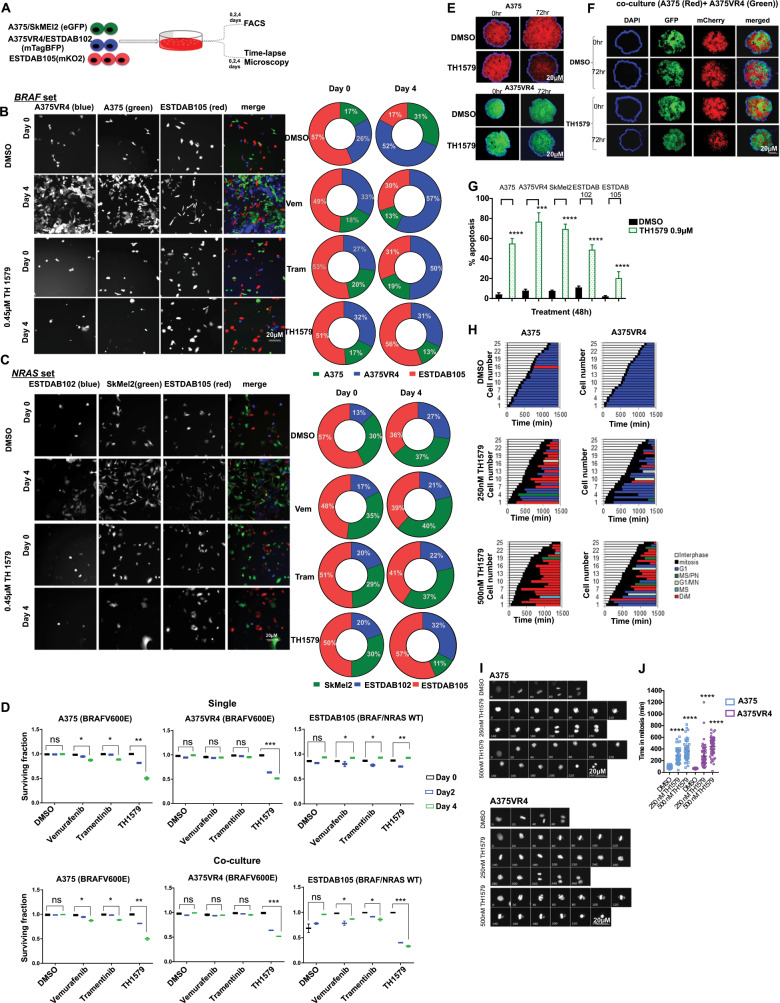
TH1579 treatment causes prolonged time in mitosis and cell death in co-culture (NRAS and BRAF sets) as well as in 3D spheroid culture of CMM cells. **a** Schematic illustration of single and co-culture experiments to compare drug sensitivity. Briefly, A375 and SkMEl2 cells were tagged with eGFP (green), A375VR4 and ESTDAB102 with mTagBFP (blue) and ESTDAB105 with mKO2 (red). Cells were then either single cultured or co-cultured as *BRAF* set (A375, A375VR4, and ESTDAB105) or *NRAS* set (SkMel2, ESTDAB102, and ESTDAB105) overnight before treatment with DMSO or BRAF inhibitor (Vemurafenib, Vem, 0.4 μM), MEK inhibitor (Trametinib, Tram, 3 nM), or MTH1 inhibitor (TH1579, 0.45 μM) for 4 days. Time-lapse microscopy was performed to detect changes in cell proliferation by measuring number of cells (DAPI count) followed by quantification using cell profiler software. The proportion of each CMM cell line (shown in **b** and **c**) was calculated as % of total number of cells day 0 (i.e., before treatment) and day 4 (i.e., following 4 days treatment), respectively. FACS was performed on day 0, 2, and 4 to measure fraction of apoptotic cells (measured as a ratio of number of labeled + AnnV + cells to total number of labeled + cells). **b** For the *BRAF* set, the BRAFi resistant A375VR4 cell approximately doubled its proportion after 4 days in control (DMSO), vemurafenib as well as trametinib treated cells. TH1579 treated co-culture showed almost no change in cell proportions day 4 compared with day 0. **c** For the *NRAF* set, TH1579 was particularly effective in stopping proliferation of SkMel2, since the proportion of SkMel2 went from 30% day 0 to 11% day 4, as compared with the proportion of control treated co-culture from 30% day 0 to 38% day 4. **d** FACS analysis shows that *BRAF* mutant and *NRAS* mutant CMM cells retain their sensitivity towards TH1579 either when cultured separately or in a co-culture system (error bars represent mean ± SD; *n* = 3, *p < 0.05, **p < 0.01, ***p < 0.001, ****p < 0.000, Student’s *t* test). Data shown as ratio of number of Annexin V labeled cells to total number of labeled cells for each cell line (i.e., fraction of survival cells) either co-cultured or as cultured separately, before treatment (day 0), and treatment day 2 and 4. **e** Representative images of A375 and A375VR4 spheroids treated with DMSO (control) or TH1579 (2 µM) for 72 h (*n* = 3). **f** Representative images of spheroid co-culturing of A375 with its vemurafenib resistant subline A375VR4 before and following DMSO (control) or TH1579 (2 µM) for 72 h (*n* = 3). **g** Treatment with TH1579 triggers cell death via induction of apoptosis. CMM cells were treated for 48 h with 0.9 µM TH1579 and analyzed by Annexin V + stain and PI + stain (FACS) (error bars represent mean ± SD; *n* = 3, *p < 0.05, **p < 0.01, ***p < 0.001, ****p < 0.000, Student’s *t* test). **h** Histone labeling followed by time-lapse microscopy analysis shows that A375 and A375VR4 CMM cells treated with lower doses of TH1579 (250 nM and 500 nM) display a significantly prolonged mitotic phase (M) followed by mitotic slippage and polynucleation (MS/PN), micronuclei (G1/MN), mitotic slippage (MS) and death in mitosis (DiM). **i** Representative Images from time-lapse experiment in **h**. **j** Quantification of of the time in mitosis in **h**. (error bars represent mean ± SD; *n* = 2, *p < 0.05, **p < 0.01, ***p < 0.001, ****p < 0.0001, Student’s *t* test).

Tumor intra- and inter-heterogeneity is common in CMM and heterogeneity presents itself as a resistance mechanism. To recapitulate this in vitro, we established a system where we labeled each cell line with a different fluorescent protein, and divided them into a “*BRAF* set” or a “*NRAS* set” (Fig. 2a). ESTDAB105 grow slower than the *BRAF/NRAS* cells and therefore the ratio was adjusted based on respective cell lines proliferation rate. After 4 days of co-culturing, the majority of the “*BRAF set*” culture consisted of the A375VR4 in the control (DMSO) group as well as after treatment with trametinib and vemurafenib (Fig. 2b). Interestingly, the proportions of the various cells following treatment with TH1579 were similar to prior treatment (Fig. 2b), indicating that TH1579 was equally potent in killing the CMM cells. After 4 days of co-culturing of the “*NRAS set*” similar proportions of CMM cells could be observed day 4 as day 0 following vemurafenib or trametinib treatment (Fig. 2c). TH1579 treatment was more effective in hindering SkMel2 proliferation as the proportion of this cell line was lower at day 4 compared with day 0 (Fig. 2c).

TH1579 was equally or more effective than vemurafenib and trametinib in killing cells in co-culture as well as individually cultured cells when analyzing surviving fractions by using FACS (Fig. 2d and Supplementary Fig. S2).

We also cultured 3D spheroids using our labeled cell lines, A375 and A375VR4, and compared the differences in response with TH1579 either when co-cultured or cultured alone. The effects were documented at 24 and 72 h, showing significant reduction of 3D spheroids independent whether co-cultured or cultured alone (Fig. 2e, f).

### TH1579 mediates loss of cell viability mainly through induction of apoptosis

To determine whether the reduced cell viability and growth inhibition was a result of apoptosis, five cell lines with different *BRAF/NRAS* mutational status were selected and treated with TH1579 for 24 and 48 h, respectively. The IC50 of the BRAFi (vemurafenib) resistant subline, A375VR4, was chosen as the concentration used. FACS analysis demonstrated that CMM cells underwent apoptosis already after 24 h (Supplementary Fig. S3a) which was further enhanced after 48 h of treatment (Fig. 2g). The induction of apoptosis after 48 h for *BRAF* (~50–75% (*p* < 0.001)) and *NRAS* mutant lines (~50–75% (*p* < 0.001)) was higher than for *BRAF/NRAS* WT cell line ESTDAB105 (25% (*p* < 0.001)) (Fig. 2g).

TH1579 treatment led to G2/M arrest in ESTDAB105 (*p* < 0.0001) (Supplementary Fig. S3b, c), followed by a significant decrease in cell proliferation as assessed by fewer colonies being formed (*p* < 0.0001) (Supplementary Fig. S3d, e). A similar G2/M arrest was also observed in the *BRAF/NRAS* WT cell line ESTDAB138 (Supplementary Fig. S3f). By using time-lapse microscopy, we observed a significant prolonged time in mitosis following TH1579 treatment in both A375 and A375VR4 cells, resulting in polynucleation and apoptosis (Fig. 2h–j, Movie MV1–MV4).

### TH1579 causes induction of ROS and DNA damage in CMM cells

**Fig. 3:**
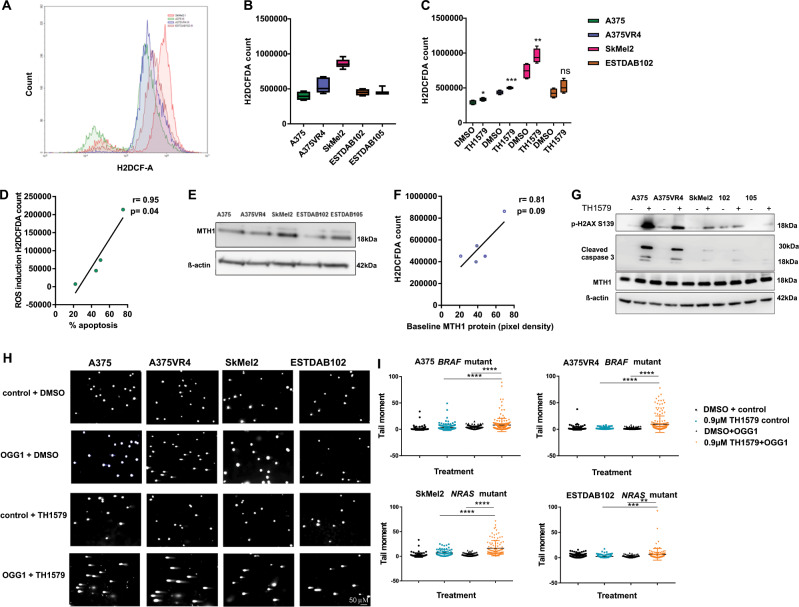
TH1579 induces ROS leading to increased DNA damage in CMM cells. **a** FACS plot showing differences in baseline ROS levels across CMM cells as measured by H2DCFA counts. **b** Quantification of **a** (error bars represent mean ± SD; *n* = 3). **c** CMM cells treated for 3 h with TH1579 (0.9 µM) displays elevated ROS levels using H2DCFA assay (error bars represent mean ± SD; *n* = 3; *p < 0.05, **p < 0.01, ***p < 0.001, ns: not significant, Student's *t* test). **d** TH1579 induced ROS (data shown in **c**) is positively correlated with percentage of apoptosis (data obtain in Fig. 2g) after drug treatment. **e** Representative image from western blot showing baseline MTH1 expression levels in CMM cells. **f** A positive trend, however not significant, is found between baseline MTH1 expression (data obtained from **e**) and ROS levels (data obtained from **b**) in CMM cells. **g** Representative image of western blot showing that 24 h treatment with 0.9 µM TH1579 (*n* = 3) increases p-H2AX signal (DNA damage marker) and cleaved caspase 3 (apoptosis marker) in CMM cells. **h** Representative images of modified comet assay in CMM cells (A375, A375VR4, and ESTDAB102) treated with TH1579 (0.9 µM) and SkMel2 treated with 0.6 µM TH1579. Comets are treated without (control) and with OGG1 to identify 8-oxodG. **i** Quantification of **h** (error bars represent mean ± SD; *n* = 3; *p < 0.05, **p < 0.01, ***p < 0.001, Student’s t test).

To investigate if ROS levels can influence CMM cells response to TH1579 treatment we measured ROS by using a H2DCFDA assay. Baseline levels of ROS demonstrated differences between the cell lines with SkMel2 having the highest basal ROS levels (Fig. 3a–b). The ROS levels were most pronouncedly induced upon short treatment (3 h) with 0.9 µM TH1579 (*p* < 0.01) in A375VR4 and SkMEl2 (Fig. 3c). There was no significant increase in ROS levels for ESTDAB105 (Supplementary Fig. S3g), which may explain why apoptosis was lower in this cell line (Fig. 2g). There was a significant correlation observed between ROS induction and induced apoptosis with *r* = 0.95 and *p* < 0.05 (Fig. 3d). There was no significant correlation between MTH1 protein levels at baseline and baseline ROS (Fig. 3f). SkMel2, one of the cell lines with high sensitivity to TH1579, also had high MTH1 protein levels (Fig. 3e).

Treatment for 24 h with TH1579 did not reduce the MTH1 protein levels (Fig. 3g), indicating that TH1579 did not inhibit the translation of the protein. TH1579 induced elevated levels of DNA damage marker (p-H2AX) in all CMM cells tested (Fig. 3g). This induction was most predominant in A375VR4 which also had a higher baseline of p-H2AX. Confirming previous findings (Fig. 2d), cleaved caspase 3 was upregulated in all cell lines, except ESTDAB105 (Fig. 3g and Supplementary Fig. S3h). TH1579 treatment also induced 8-oxo-dG incorporation in CMM cells as measured with a modified comet assay (Fig. 3h, i).

### Silencing MTH1 alone exhibits cytotoxic effects in most CMM cell lines

**Fig. 4:**
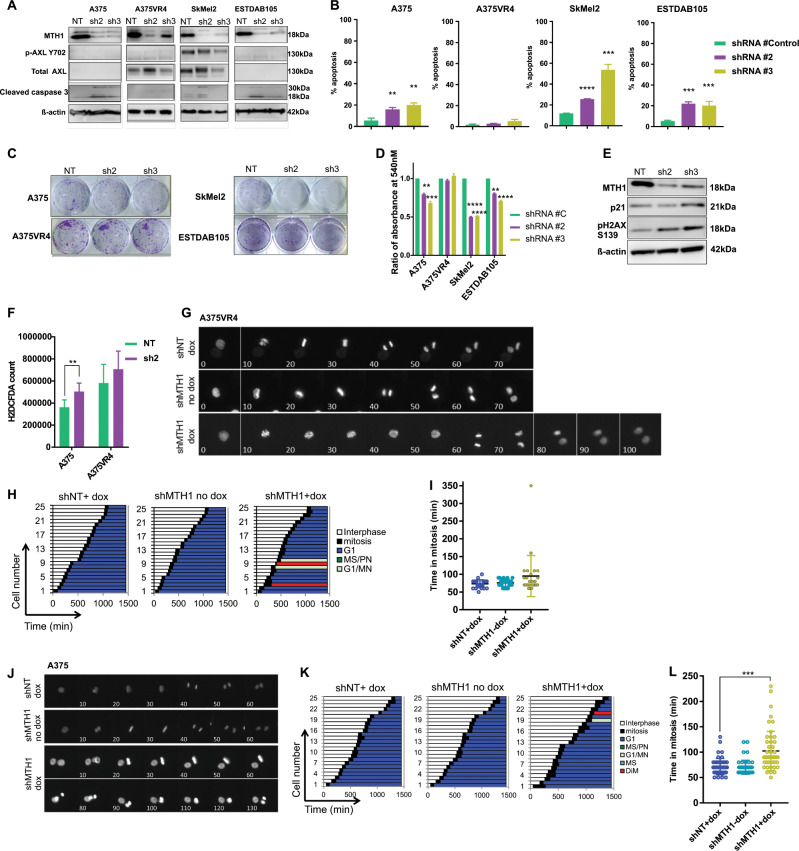
Knockdown of MTH1 in CMM cells, excluding BRAF inhibitor resistant subline A375VR4, sensitizes cells towards cell death. **a** A representative image from western blot of MTH1 knockdown in CMM cell lines. Two different shRNAs were used (sh2 and sh3) and compared with control (NT). Knockdown of MTH1 induces cleaved caspase 3 in all cell lines tested except A375VR4 (*n* = 2 independent experiments). **b** shMTH1 induces apoptosis measured by FACS (Annexin V) in A375, SkMel2, and ESTDAB105, but not in A375VR4 CMM cells. (error bars represent mean ± SD; *n* = 3; *p < 0.05, **p < 0.01, ***p < 0.001, Student's *t* test). **c** Representative images from clonogenic survival following shRNA MTH1 knockdown (sh2 and sh3) or control (NT) in CMM cell lines. **d** Quantification of **c** by measuring absorbance at 540 nm as a readout. (error bars represent mean ± SD; *n* = 3; *p < 0.05, **p < 0.01, ***p < 0.001, Student's *t* test). **e** Knockdown of MTH1 (sh2, sh3) in A375VR4 cells followed by western blot shows that the knockdown can cause induction of DNA damage (p21 and p-H2AX S139) for both shRNA sequences tested when compared with the nontargeting control shRNA. (western blot is a representative image of *n* = 2). **f** ROS levels measured by H2DCFA assay following MTH1 knockdown (sh2) or control (NT) in A375 cells and A375VR4. (error bars represent mean ± SD; *n* = 3; *p < 0.05, **p < 0.01, ***p < 0.001, Student's *t* test). **g** Representative images from time-lapse microscopy following doxycycline-induced knockdown (shMTH1 dox) of MTH1 or control (shNT dox and shMTH1 no dox) in A375VR4 cells. **h**, **i** Quantification of **g**. MTH1 knockdown (doxycycline-induced shRNA) in A375VR4 cells show no change of time in mitosis (error bars represent mean ± SD; *n* = 2, *p < 0.05, **p < 0.01, ***p < 0.001, ****p < 0.0001). **j** Representative images from time-lapse microscopy following doxycycline-induced knockdown (shMTH1 dox) of MTH1 or control (shNT dox and shMTH1 no dox) in A375 cells. **k**, **l** Quantification of **j**. MTH1 knockdown (doxycycline-induced shRNA) in A375 cells induce significantly prolonged time in mitosis (*p* < 0.01) (error bars represent mean ± SD; *n* = 2; ***p < 0.001, Student's *t* test).

To determine the importance of MTH1 as a target in CMM, we knocked down MTH1 using an inducible shRNA based approach. MTH1 knockdown caused induction of cleaved caspase 3, significant induction of cell death and reduced ability to form colonies in three out of four cell lines tested, while there was no effect on A375VR4 (*p* < 0.1) (Fig. 4a–d). Surprisingly, despite no effect on cell survival, the MTH1 knockdown in A375VR4 induced elevated DNA damage markers (Fig. 4e), showed a tendency to increase ROS (Fig. 4f) and a slight prolonged time in mitosis (Fig. 4g–i, Movie MV5, MV6). As observed with TH1579, MTH1 knockdown in A375 cells significantly prolonged time in mitosis (Fig. 4j–l) and significantly elevated ROS levels (Fig. 4f).

### CMM cells with high AXL are more sensitive to TH1579 treatment

**Fig. 5:**
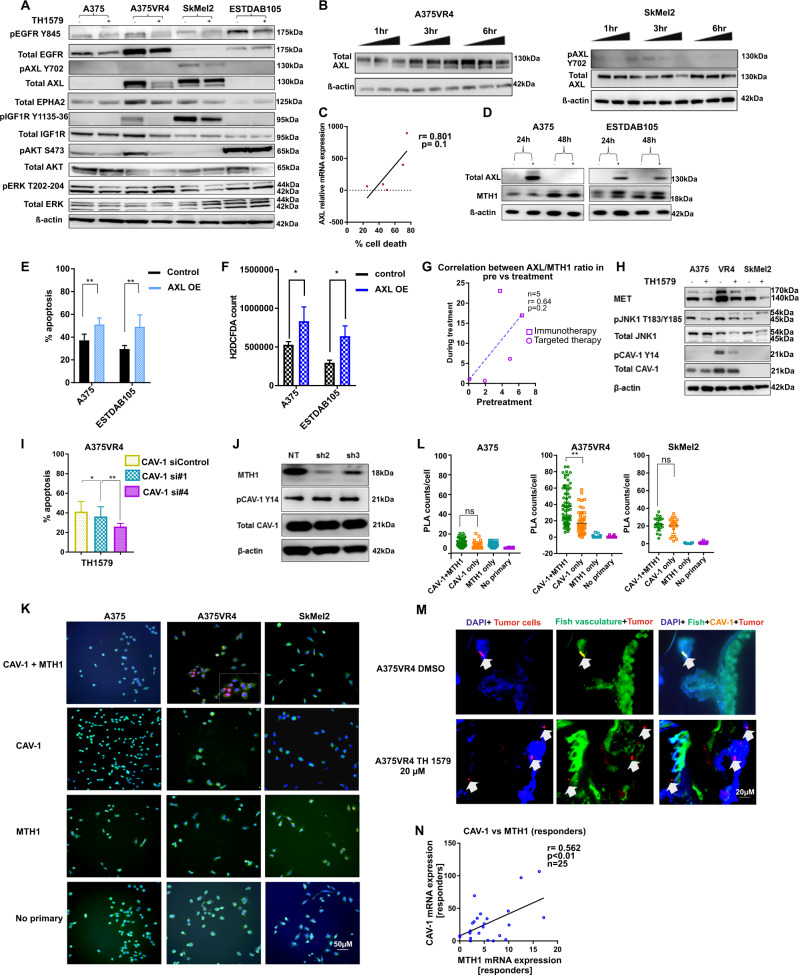
AXL and CAV-1 mediate sensitivity of CMM cells towards TH1579. **a** Representative image of western blot without (−) and with (+) 24 h treatment of 0.9 µM TH1579 in CMM cells. **b** Short-term (1, 3, and 6 h) treatment with TH1579 (0.3 and 0.9 µM) downregulates AXL in AXL overexpressing CMM cells as seen in representative image of western blot. **c** Cells with higher AXL mRNA expression have tendency to be more sensitive to TH1579. Data are obtained from Figs. 2g and 5a and plotted as correlation between AXL expression (normalized to loading control) and % apoptosis (Annexin V staining, FACS). **d** AXL overexpression was done in CMM cells with endogenous low AXL. The image is a representative western blot from *n* = 2 independent experiments. **e** Overexpression of AXL triggers sensitivity towards TH1579. Overexpression was done in CMM cells with low AXL (A375, ESTDAB105) for 24 h followed by 24 h treatment with 0.9 µM TH1579 and apoptosis measured by using FACS staining for Annexin V+, PI+. (error bars represent mean ± SD; *n* = 3; *p < 0.05, **p < 0.01, ***p < 0.001, Student's *t* test). **f** ROS measurement by H2DCFDA assay confirms that ROS is induced in both cell lines upon AXL overexpression (O.E) compared with control cells. (error bars represent mean ± SD; *n* = 3; *p < 0.05, **p < 0.01, ***p < 0.001, Student's *t* test). **g** Ratio of AXL and MTH1 expression correlate positively, although not significant (*r* = 0.64, *p* = 0.2) in CMM patients on comparing matched pretreatment samples to those taken during treatment (immunotherapy or targeted therapy) (*n* = 5). **h** Representative western blot image of *n* = 2 independent experiments. CAV-1 which is highly expressed in A375VR4 is downregulated after 24 h treatment with 0.9 µM TH1579 compared with control treated cells. Also MET and JNK1 (MAPK8) are downregulated compared with control cells. **i** Knockdown of CAV-1 (siRNA#1 and #4) decreases TH1579 sensitivity as analyzed by Annexin V staining using FACS in A375VR4 CMM cells (error bars represent mean ± SD; *n* = 3; *p < 0.05, **p < 0.01, ***p < 0.001, Student's *t* test). **j** shMTH1 (sh2 and sh3) does not reduce CAV-1 expression in A375VR4. Contrary to the inhibitor, knockdown of MTH1 alone does not decrease CAV-1 expression levels as seen by western blot (Data shown as a representative western blot image, *n* = 2). **k** Proximity Ligation Assay (PLA) shows CAV-1 and MTH1 interact in A375VR4. Analysis by PLA indicates that MTH1 and CAV-1 interacts only in A375VR4 and not in A375 or SKMel2. Data shown as a representative image, *n* = 2. **l** Quantification of (K) (error bars represent mean ± SD; *n* = 2; *p < 0.05, **p < 0.01, ***p < 0.001, Student's *t* test). **m** Treatment with TH1579 (20 µM) decreases expression of CAV-1 in an A375VR4 zebrafish disease model as seen by IF. **n** CAV-1 expression correlates to MTH1 expression in CMM patients both in responders to targeted and immunotherapy (*n* = 25). Sample set previously used in [44] (*p < 0.05, Student's *t* test).

Since it is known that ROS activates RTKs [32, 33], we wanted to investigate if RTKs play a role in TH1579 mode of action. Based on the results obtained from a pRTK array screen upon treating A375, A375VR4, SkMel2, and ESTDAB105 for 24 h with 0.9 µM TH1579 (data not shown) and a previously published study [31], we decided to further investigate TH1579 induced effects on AXL, EPHA2, EGFR, IGF1R, MET and some downstream effectors. AXL and EPHA2 (A375VR4 and SkMel2), EGFR and IGF1R (A375 and A375VR4), MET were downregulated (Fig. 5a and Supplementary S4a) upon exposure to TH1579. Downstream effectors JNK1 and AKT were downregulated after 24 h exposure to TH1579 in A375 and A375VR4 and after 48 h  in SkMEl2, whereas pERK was significantly downregulated only in A375 (Fig. 5a–h and Supplementary Fig. S4a, b). We focused on AXL since it has been suggested to have an impact on both *BRAF* targeted and DNA damaging therapies [20, 24]. A consistent downregulation of AXL and AKT expression was observed in longer term cultures (48 h) of both A375VR4 and SkMel2 (Supplementary Fig. S4c).

To determine if the change of AXL following TH1579 treatment was an early event, A375VR4 and SkMel2 were exposed to TH1579 for 1, 3, and 6 h. In A375VR4, a decrease of AXL was observed at 6 h, whereas in SkMel2 there was a reduction of AXL already at 3 h. (Fig. 5b and Supplementary Fig. S4d). These changes were most likely occurring posttranslationally, since mRNA levels of AXL was unchanged (Supplementary Fig. S4e). Downregulation of AXL by TH1579 was also confirmed in CMM xenografted zebrafish models (Supplementary Fig. S5a). Using an in vitro kinase assay, we showed that TH1579 did not inhibit AXL activity (Supplementary Fig. S5b), indicating that the downregulation of AXL is a downstream effect of TH1579 and not a direct target of the drug.

Correlation analysis revealed that there was a positive trend, although not significant, between basal AXL mRNA expression and apoptosis induction by TH1579 (*r* = 0.801, *p* = 0.1) (Fig. 5c). To confirm that high levels of AXL make the cells more prone to respond to TH1579 treatment, we overexpressed AXL in cell lines with low AXL expression (Fig. 5d). Indeed, AXL induction significantly increased inhibitor mediated cell death in both A375 and ESTDAB105 (*p* < 0.01) (Fig. 5e), highlighting that AXL may play an important role in determining the sensitivity of CMM cells towards TH1579.

Since ROS induction in turn induces AXL in a ligand independent fashion [34], we next sought out to find if AXL overexpression can induce ROS levels and thereby make the cells more sensitive to a MTH1 inhibitor. We observed that AXL overexpression increased ROS levels by around 50% in both A375 and ESTDAB105 (Fig. 5f). Interestingly, a positive trend, though not significant (*p* = 0.2) was observed between the ratio of AXL and MTH1 in our matched clinical samples from CMM patients (Fig. 5g).

A network analysis (http://www.networkanalyst.ca/) was performed on the RTKs whose expression was altered upon 24 h treatment with TH1579. The results indicated that different gene ontologies were affected including regulation of apoptosis, stress response, cell cycle, and cell migration. Furthermore, KEGG analysis showed that CMM was the most significantly altered disease associated with changes in RTKs and MTH1 (Supplementary Fig. S6).

### Inhibition of CAV-1 leads to decreased response to TH1579 in BRAF inhibitor resistant cell line A375VR4

From previous studies in our lab [27], we know that A375VR4 has high expression of CAV-1 mRNA (Supplementary Table S2). TCGA analysis shows that AXL, MTH1, and CAV-1 have a higher alteration frequency for increased mRNA expression (Supplementary Fig. S7a). Our cohort of samples from targeted and immunotherapy (*n* = 32) show slightly elevated median levels of AXL expression in nonresponders to targeted therapy (Supplementary Fig. S7b). Furthermore, TCGA analysis revealed that CAV-1 mRNA expression is increased in patients with disease stage I-IV vs stage 0 (Supplementary Fig. S7c). However, we did not find any correlation between AXL-MTH1 and CAV-1-MTH1 with regards to the stage of the disease (Supplementary Fig. S7d, e).

Interestingly, we observed that both p-CAV-1 and total CAV-1 expression was reduced in A375VR4 after TH1579 treatment (Fig. 5h). Using CETSA, we observed no direct target engagement of TH1579 to CAV-1 (Supplementary Fig. S8a), indicating that the observed reduction of CAV-1 levels is a downstream effect of the treatment. Interestingly, when CAV-1 was downregulated, the TH1579-mediated apoptosis in A375VR4 was significantly abolished (Fig. 5i). However, knocking down MTH1 alone in A375VR4 did not downregulate CAV-1 (Fig. 5j) while silencing of CAV-1 led to downregulation of both AXL and MTH1, which may explain the loss of effect by TH1579. (Supplementary Fig. S8b).

Co-IP experiments showed a weak interaction between CAV-1 and MTH1 in A375VR4 (Supplementary Fig. S9a). In contrast, no protein–protein interaction between MTH1-AXL was found (Supplementary Fig. S9b, c). To further verify our observation, we performed PLA analysis, confirming that CAV-1 interacted with MTH1 (Fig. 5k, l). Downregulation of CAV-1 expression in our in vivo zebrafish model further corroborated that TH1579 inhibits CAV-1 expression (Fig. 5m). We also observed a positive correlation between MTH1 and CAV-1 expression in clinical samples taken pretreatment (Fig. 5n).

### Combining BRAF inhibitors to TH1579 further sensitizes *BRAF* mutant cells

**Fig. 6:**
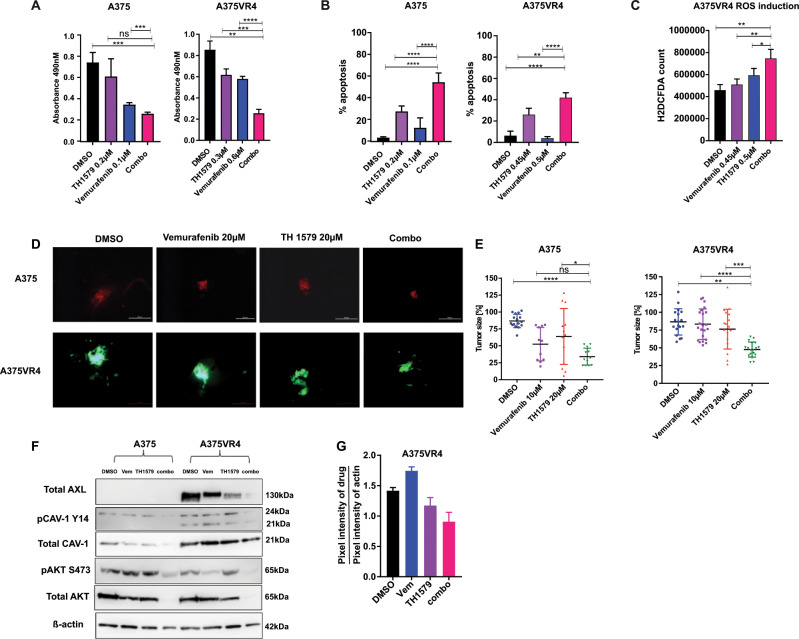
Combination treatment of TH1579 with vemurafenib (PLX4032) further sensitizes BRAF mutant CMM cells both in vitro and in vivo. **a** 72 h treatment of *BRAF* mutant cells A375 and A375VR4 with the combination TH1579 (0.2 µM) and vemurafenib (0.1 µM) leads to further loss of cell viability as measured by an MTS assay when compared with either drug alone (error bars represent mean ± SD; *n* = 3; *p < 0.05, Student's *t* test). **b** The loss of cell viability observed in **a** translates into apoptosis mediated cell death which is further enhanced by the drug combination (treatment done for 48 h) as analyzed by FACS and Annexin V staining (error bars represent mean ± SD; *n* = 3; *p < 0.05, Student’s *t* test). **c** Combining vemurafenib with TH1579 significantly induces ROS levels (treatment done for 3 h) when compared with either drug alone (error bars represent mean ± SD; *n* = 3; *p < 0.05, Student's *t* test). **d** Representative images taken by microscopy to show A375 (stably expressing dTomato) and A375VR4 (stably expressing eGFP) tumors in zebrafish disease model after 72 h drug exposure with DMSO, vemurafenib (20 µM), TH1579 (20 µM) or the combination. **e** Individual tumor sizes from zebrafish embryos were analyzed after lysing zebrafish and measure luminescence. Data presented as % tumor size compared with the median tumor size calculated for that group (error bars represent mean ± SD; *n* = 3; *p < 0.05, **p < 0.01, ***p < 0.001, ****p < 0.0001, Student's *t* test). **f**, **g** CAV-1, AXL, and AKT are most efficiently downregulated by combination treatment following 48 h treatment. Representative western blot image (**f**) and densiometric quantification of the western blot (**g**) (data shown as mean ± SD, *n* = 2).

To investigate whether the combination of BRAFi and TH1579 could further potentiate the killing of *BRAF* mutant CMM cells we used a proliferation assay. We observed that the combination of vemurafenib or dabrafenib with TH1579 significantly reduced cell proliferation and induced cell death (*p* < 0.01) in A375, A375VR4, and ESTDAB049 cell lines compared with either treatment alone (Fig. 6a, b and Supplementary Fig. S10). We observed that the combination treatment of vemurafenib and TH1579 induced more ROS than either treatment alone (Fig. 6c and Supplementary Fig. S10). Furthermore, the combination treatment reduced the tumor size in the zebrafish by 3.5- and 4-fold in A375 and A375VR4, respectively (*p* < 0.05) (Fig. 6d, e). Interestingly, the combination downregulated AXL, CAV-1, and AKT when compared with single treatment arms or DMSO control in both cell lines (Fig. 6f, g), which may explain the improved efficacy observed in the combination treatment compared with monotherapy with TH1579.

## Discussion

**Fig. 7:**
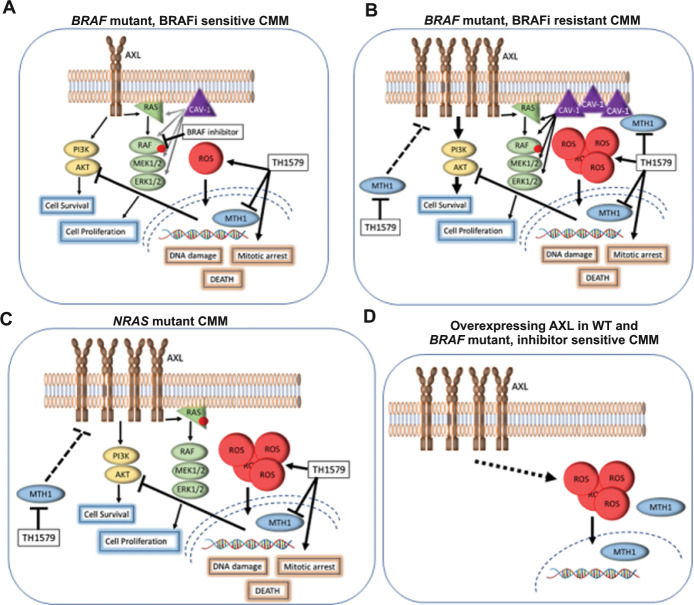
Schematic illustration how TH1579 downregulates AXL and CAV-1 expression and induces ROS, leading to DNA damage and cell death. **a** In *BRAF* mutant CMM, TH1579 treatment inhibits MTH1, elevates ROS, causes a mitotic arrest, DNA damage and reduced AKT signaling. Combining BRAF inhibitor vemurafenib and TH1579 results in additive/synergistic responses by affecting several pathways leading to apoptosis and cell death. **b** BRAF inhibitor resistant CMM have upregulated levels of AXL and CAV-1, resulting in overactivated MAPK signaling overriding the effect of BRAF inhibitor. In addition to the effects of TH1579 observed in *BRAF*-mutated CMM, TH1579 downregulates AXL and CAV-1 levels, resulting in improved efficacy of BRAF inhibitor treatment. **c** *NRAS* mutated CMM has upregulated AXL compared with *BRAF* mutated and WT CMM and TH1579 treatment reduces the level of AXL, hinder AKT signaling, induces ROS, causes DNA damage, mitotic arrest and cancer cell death. **d** By overexpressing AXL in WT and *BRAF*-mutated CMM, increased ROS levels and improved efficacy following TH1579 treatment was observed.

The significance of ROS for the treatment effect as well as resistance development cannot be underestimated in CMM- a tumor type heavily laden with ROS. It has been postulated that DNA repair proteins play a vital role in maintaining overall cellular homeostasis by maintaining ROS levels [35]. One such player is MTH1, which cancer cells often use as a major defense mechanism to eliminate elevated intracellular ROS. We now identify TH1579, a potent inhibitor of MTH1, as a highly efficacious drug to treat CMM by not solely inhibiting MTH1, but also through downregulation of key proteins in BRAFi resistance pathways, like AXL and CAV-1. These changes are concomitant with increased incorporation of damaged nucleotides, increase in intracellular ROS and mitotic arrest (Fig. 7).

MTH1 loss has previously been shown to selectively eradicate cells with high levels of RAS and ROS [36]. Our results corroborate that excessive ROS led to elevated apoptosis following TH1579 treatment, but the effect was independent of *BRAF*/*NRAS* mutations (Figs. 1 and 2 and Supplementary Fig. S2). This was in line with previous findings in CMM mice xenograft models [37] and may be explained by a general high basal ROS level detected in WT as well as *BRAF/NRAS* mutated CMM cells (Fig. 3b).

Recent studies show on one hand that MTH1 is an important target for cancer [14, 15, 37], whereas on the other hand some studies claim MTH1 inhibition to be nontoxic, thereby questioning the anticancer therapeutic potential of MTH1i [38, 39]. We reported that mitotic arrest, elevated ROS and 8-oxodGTP are important for the efficacy of TH1579 and that noncytotoxic MTH1i can show synergistic effects when combined with mitotic arrest agents [40]. Emerging evidence connect mitotic arrest with ROS. For instance, it has been shown that the level of ROS changes with cell cycle, where ROS is increased during mitosis and a mitotic arrest further enhances ROS levels [41]. Whether MTH1 directly alter ROS levels and plays a role during mitosis is still under debate. Here, we observe that knockdown of MTH1 in A375, but not in A375VR4, significantly prolonged time in mitosis with a concomitant increase in ROS levels resulting in cell death (Fig. 4), thus suggesting that MTH1 may directly influence ROS level and play a role in mitosis in A375 cells. One explanation to the discrepancy in A375 and A375VR4 MTH1 knockdown could be that the knockdown in A375VR4 cells was not as successful as in the other CMM cells. Other plausible explanations could be that some dNTP sanitizing enzymes may compensate for the loss in MTH1 activity [42] or that CAV-1 silencing is essential to stimulate cytotoxic effects in these cells as shown in this study (Fig. 5).

Limited duration of efficacy of therapy modules have often been attributed to upregulation of RTK signaling which is caused by the high phenotypic plasticity of CMMs. We have shown that TH1579 is able to downregulate several key RTKs associated with therapy resistance including AXL (Fig. 5 and Supplementary Figs. S4 and S5). Moreover, we have also shown that CMM cells with high AXL are more sensitive to TH1579 inhibitor. Recent studies indicate that CAV-1 has several other functions beyond protein transport like its role in signal transduction, tumor progression and tumor regression and also that KRAS inhibits MTH1 function through CAV-1 [43]. We have confirmed a weak interaction between CAV-1 and MTH1 in BRAFi resistant CMM cells (Fig. 5 and Supplementary Fig. S9). Our results suggest a novel mode of regulation involving CAV-1 and MTH1 where CAV-1 positively modulates TH1579 cytotoxicity (Fig. 5 and Supplementary Figs. S8 and 9).

The current strategy of treatment employing targeted therapy for CMM patients with *BRAF* mutation is the combination of BRAFi and MEKi [1]. To this end, we have also highlighted that combining TH1579 together with BRAFi might be an attractive therapy regime of choice since our study showed that the combination could enhance cell killing both in vitro and in vivo.

Herein, we show that CMM cells are sensitive to TH1579, independent of *BRAF/NRAS* mutational status. We unveil important correlations between AXL and CAV-1, highlighting their role in determining overall sensitivity of CMM cells towards TH1579 treatment. Importantly, we are the first one to report that induction of AXL is able to induce cellular ROS levels. Lastly, we show that combining BRAFi with TH1579 was more potent than the single drugs. TH1579 (Karonudib) is presently investigated in a clinical Phase 1 trial in patients with advanced solid malignancies (www.clinicaltrial.gov//MASTIFF). We envision TH1579 as a promising alternate therapy for CMM patients where AXL and CAV-1 may be potential predictive biomarkers. Combining TH1579 with other drugs would enhance the clinical applicability of TH1579.

## Supplementary information

Supplementary movie MV1

Supplementary movie MV2

Supplementary movie MV3

Supplementary movie MV4

Supplementary movie MV5

Supplementary movie MV6

Supplementary material and method

Supplementary table

Supplementary figure legends

Supplementary movie legends

Supplementary figure S1

Supplementary figure S2

Supplementary figure S3

Supplementary figure S4

Supplementary figure S5

Supplementary figure S6

Supplementary figure S7

Supplementary figure S8

Supplementary figure S9

Supplementary figure S10
