# Supplementary material for: AXL and CAV-1 play a role for MTH1 inhibitor TH1579 sensitivity in cutaneous malignant melanoma
Source: Cell Death Differ. 2020 Jan 9;27(7):2081–98. doi: 10.1038/s41418-019-0488-1 (PMC7308409; doi:10.1038/s41418-019-0488-1)
Supplement: Supplementary file 7 — Supplementary material and method [file 41418_2019_488_MOESM7_ESM.docx]

| **Antibodies** | **Source** | **Catalog number** |
| --- | --- | --- |
| pEGFRY845 | Cell Signaling Technology | #6963 |
| Total EGFR | Cell Signaling Technology | #4267 |
| Total MET | Cell Signaling Technology | #8918 |
| pAXLY702 | Cell Signaling Technology | #5724 |
| Total AXL | Cell Signaling Technology | #8661 |
| Total AXL | Abcam | #ab89224 |
| pIGF1RY1135/36/ IRβ Y1150/1151 | Cell Signaling Technology | #3024 |
| Total IGF1Rβ | Cell Signaling Technology | #9750 |
| pJNK1T183/Y185 | Cell Signaling Technology | #4668 |
| Total JNK1 | Cell Signaling Technology | #9252 |
| pEPHA2S897 | Cell Signaling Technology | #6347 |
| Total EPHA2 | Cell Signaling Technology | #6997 |
| pAKTS473 | Cell Signaling Technology | #4060 |
| Total AKT | Cell Signaling Technology | #4691 |
| p- p44/42 MAPK (ERK1/2) T202/Y204 | Cell Signaling Technology | #9106 |
| p44/42 MAPK (ERK1/2) | Cell Signaling Technology | #9102 |
| pCAV1Y14 | Cell Signaling Technology | #3251 |
| Total CAV1 | Cell Signaling Technology | #3267 |
| Total CAV1 | Abcam | #ab17052 |
| pp53S15 | Cell Signaling Technology | #9284 |
| Total p53 | Santa Cruz | sc-126 |
| MTH1 | Abcam | #ab200832 |
| MTH1 | Novus Biologicals | #NB 100-109 |
| Cleaved caspase-3 | Cell Signaling Technology | #9661 |
| P21 | BD biosciences | #610234 |
| p-H2AXS39 | Cell Signaling Technology | #9718 |
| Phallodin | Thermo Fischer Scientific | #A12379 |
| β-actin (HRP labeled) | Cell Signaling Technology | #12262 |
| Secondary rabbit (HRP labeled) | Cell Signaling Technology | #7074 |
| Secondary mouse (HRP labeled) | Cell Signaling Technology | #7076 |
| Secondary Mouse (Alexa Flor 568) | Abcam | ab175473 |
| Secondary rabbit (Alexa Flor 555) | Abcam | ab150074 |
| Secondary biotin | Cell Signaling Technology | #7727 |
| Andy Flour 647, Annexin V | BioCat | #A038-GC |

**Chemicals**

| Vemurafenib (PLX4032) | Selleckchem | #S1267 |
| --- | --- | --- |
| Trametinib (GSK1120212) | Selleckchem | #S2673 |
| Dabrafenib (GSK2118436) | Selleckchem | #S2807 |
| DMEM-Glutamax | Thermo Fischer Scientific | # 10566-016 |
| MEM | Thermo Fischer Scientific | # 11095-080 |
| RPMI-1640 | Thermo Fischer Scientific | # 21875091 |
| Lipofectamine 2000 | Invitrogen | #11668027 |
| Opti-MEM | Thermo Fischer Scientific | # 31985070 |

**Cell lines, plasmids**

| Human: A375 | ATCC |  |
| --- | --- | --- |
| A375VR4 | (30) |  |
| A375PR1 | (30) |  |
| Human: SkMel24 | ATCC |  |
| Human: SkMel28 | ATCC |  |
| Human: SkMel2 | ESTDAB |  |
| Human: ESTDAB102 | ESTDAB |  |
| Human: ESTDAB149 | ESTDAB |  |
| Human: ESTDAB105 | ESTDAB |  |
| Human: ESTDAB138 | ESTDAB |  |
| Human: 159-PRE | This study | FNA from CMM patient (*BRAF* wild-type ) |
| A375-eGFP | This study | N/A |
| A375VR4-mTagBFP | This study | N/A |
| SkMel2-eGFP | This study | N/A |
| ESTDAB102-mTagBFP | This study | N/A |
| ESTDAB105-mKO2 | This study | N/A |
| A375 NT/ shMTH1 | This study | N/A |
| A375VR4 NT/ shMTH1 | This study | N/A |
| SkMel2 NT/ shMTH1 | This study | N/A |
| ESTDAB105 NT/ shMTH1 | This study | N/A |
| pcDNA3.1(+)/Luc2=tDT | Addgene | #32904 |
| pLenti CMV puro Luc (w168-1) | Addgene | #17477 |
| pBluescript PMS2 | Addgene (35) | #16457 |
| pX1 | Addgene (36) | #46848 |
| pIRES-EGFP- puro | Addgene | #45567 |
| pIRES –puro2 AXL | Addgene (37) | #65627 |
| pLenti-CMV hygro DEST plasmid | Addgene | #17454 |
| pENTR1A no ccDB | Addgene | #17398 |
| mTagBFP2-pBAD | Addgene | # 54572 |
| mKO2-N1 | Addgene | # 54625 |

**Commercial kits**

| Annexin V-FLOUS staining | Sigma Aldrich | #11858777001 |
| --- | --- | --- |
| AXL kinase | Promega | #V3961 |
| ADP Glow Assay | Promega | #V9101 |
| PLA | Sigma -Aldrich | #DUO82008 |
| Plasmid DNA purification | Qiagen | #12125 |

**siRNA and shRNA sequences**

| **siCAV1 #1** | CUAAACACCUCAACGAUGA |
| --- | --- |
| **siCAV1 #2** | GCAAAUACGUAGACUCGGA |
| **siCAV1 #3** | GCAGUUGUACCAUGCAUUA |
| **siCAV1 #4** | GCAUCAACUUGCAGAAAGA |
| **shMTH1 NT** | GGAACTAGCATACGTAAGTAA |
| **shMTH1 #2** | CGAGTTCTCCTGGGCATGAAA |
| **shMTH1 #3** | CGACGACAGCTACTGGTTT |
