# Supplementary material for: AXL and CAV-1 play a role for MTH1 inhibitor TH1579 sensitivity in cutaneous malignant melanoma
Source: Cell Death Differ. 2020 Jan 9;27(7):2081–98. doi: 10.1038/s41418-019-0488-1 (PMC7308409; doi:10.1038/s41418-019-0488-1)
Supplement: Supplementary file 8 — Supplementary table [file 41418_2019_488_MOESM8_ESM.docx]

**Table S1: TH1579, vemurafenib and trametidin IC50 values of CMM cells.** Table showing inhibitory concentration at 50% (IC50) values for TH1579, BRAF inhibitor vemurafenib or MEK1 inhibitor trametinib in CMM cells with different *BRAF/NRAS* mutational background.
(*BRAF mutant* vemurafenib resistant cells are marked in red)

| **Cell line** | **Mutational  status** | **TH1579**  **IC50 (µM)** | **Vemurafenib  (PLX 4032)**  **IC50 (µM)** | **Trametinib**  **IC50 (µM)** |
| --- | --- | --- | --- | --- |
| A375 | BRAF | 0.32 | 0.16 | 0.0013 |
| A375VR4 | BRAF | 0.87 | 3.1 | 3.16 |
| A375PR1 | BRAF | 1.16 | 3.3 |  |
| SKMel24 | BRAF | 1.40 | 4.81 | 1.16 |
| SKMel28 | BRAF | 1.06 | 4.97 | 0.031 |
| SKMel2 | NRAS | 0.23 | 5.23 | 0.020 |
| ESTDAB102 | NRAS | 0.43 | 9.5 | 0.010 |
| ESTDAB149 | NRAS | 0.32 |  |  |
| ESTDAB105 | WT | 0.40 | 19 | 0.0004 |
| ESTDAB138 | WT | 0.33 | 2.0 | 0.0018 |

**Table S2: mRNA levels of selected RTKs, CAV-1, TP53 and MTH1 in CMM cell lines.** Table showing the baseline mRNA levels of selected RTKs, TP53, CAV-1 and MTH1 in CMM cell lines

| **Cell lines** | **MTH1** | **EPHA2** | **IGF1R** | **EGFR** | **AXL** | **MET** | **CAV-1** | **TP53** |
| --- | --- | --- | --- | --- | --- | --- | --- | --- |
| A375 | 34 | 48 | 1770 | 27 | 5 | 226 | 9 | 595 |
| A375VR4 | 23 | 2194 | 966 | 670 | 896 | 560 | 129 | 869 |
| SkMel24 | 18 | 2254 | 436 | 486 | 917 | 204 | 102 | 1194 |
| SkMel28 | 14 | 48 | 1389 | 68 | 4 | 191 | 10 | 994 |
| A375PR1 | 35 | 274 | 1565 | 101 | 371 | 531 | 34 | 695 |
| SkMel2 | 24 | 629 | 770 | 34 | 350 | 297 | 1 | 456 |
| ESTDAB102 | 25 | 332 | 1796 | 337 | 54 | 613 | 0 | 626 |
| ESTDAB105 | 11 | 1434 | 1591 | 295 | 61 | 157 | 32 | 3104 |
