# Supplementary material for: AXL and CAV-1 play a role for MTH1 inhibitor TH1579 sensitivity in cutaneous malignant melanoma
Source: Cell Death Differ. 2020 Jan 9;27(7):2081–98. doi: 10.1038/s41418-019-0488-1 (PMC7308409; doi:10.1038/s41418-019-0488-1)
Supplement: Supplementary file 9 — Supplementary figure legends [file 41418_2019_488_MOESM9_ESM.docx]

**Figure S1: CMM cells are sensitive to TH1579**

**(A)** High MTH1 mRNA expression affects the disease free survival in CMM patients as seen by data analyzed from TCGA (n=403, p= 0.053). **(B)** CMM cells are able to form tumors *in vivo* in zebrafish xenograft disease models. The CMM cells shown in these images are stably expressing tdTomato and luciferase and the grafted tumor is indicated by the red signal. **(C)** Images of zebrafish embryos showing no toxicity after 72 h of treatment with vemurafenib or TH1579 in the tested dose range (2.5 µM-20 µM for Vemurafenib and 10 µM- 50 µM for TH1579).

**Figure S2: NRAS mutant cells are most sensitive to TH1579**

**(A)** NRAS mutant CMM cells (SkMel2 and ESTDAB102) either when co-cultured with WT cells (NRAS set) or cultured separately display similar sensitivity towards TH1579 as shown by FACS analysis (Annexin staining), but ESTDAB102 was less sensitive to vemurafenib and trametinib when this cell line was co-cultured with SkMEl2 and ESTDAB105. Data presented as surviving fraction which is calculated as ratio of AnnV+ labeled cells to total number of labeled cells. **(B)** Growth curves for all CMM cells used in this experiment showing their growth patterns over 4 days when exposed to vemurafenib , trametinib or TH1579 (at different concentrations as stated in the figure). Data shown as growth ratio which is normalized to day 0, i.e. amount cells day 0 is set as 1. (error bars represent mean ± SD; n = 2;*p < 0.05, **p<0.01, ***p<0.001, Student's t test).

**Figure S3: CMM cells are sensitive to TH1579 independent of BRAF/NRAS mutational status**

**(A)** FACS analysis (Annexin V staining) shows TH1579-induced apoptosis following 24 h treatment compared to control (DMSO) treated CMM cells. (error bars represent mean ± SD; n = 3; *p < 0.05, **p<0.01, ***p<0.001, Student's t test). **(B)** CMM WT cells, ESTDAB105, undergo G2/M cell cycle arrest upon treatment with TH1579 (0.9 µM) for 48 h as evaluated by cell cycle analysis. **(C)** Quantification of (B). Data shown as (error bars represent mean ± SD; n = 3; *p < 0.05, Student's t test). **(D)** Representative image of clonogenic survival in ESDTAB105 cell treated with TH1579 (0.9 µM) or control cells (DMSO). **(E)** Quantification of (D) (error bars represent mean ± SD; n = 3;*p < 0.05, **p<0.01, ***p<0.001, Student's t test). **(F)** TH1579 induces G2/M cell cycle arrest in another BRAF/NRAS WT cell line ESTDAB138 as evaluated by cell cycle analysis (error bars represent mean ± SD; n = 4; *p < 0.05, Student's t test). **(G)** ROS levels in ESTDAB105 with *BRAF/NRAS* WT mutational background remains unaltered upon treatment with TH1579 (0.9 µM, 3 h). **(H)** Pixel intensity of cleaved caspase 3 induced after treatment with TH1579 (0.9 µM, 24 h) normalized to actin (error bars represent mean ± SD; n = 3; *p < 0.05, **p<0.01, ***p<0.001, Student's t test).

**Figure S4: Quantification of western blot bands as a ratio of fold change between treatment/ DMSO.** **(A)** Densitometric analysis of western blot showing effect of 24h TH1579 (0.9 µM) treatment on selected RTKs, CAV-1 and downstream effectors (data normalized to β−actin). **(B)** Densiometric analysis of western blot showing effect of 24h TH1579 (0.9 µM) treatment on MET, JNK1 and CAV-1 (n=2) (data normalized to β−actin) (error bars represent mean ± SD; n = 2; ***p<0.001, **p<0.01, *p < 0.05, Student's t test).. **(C)** Representative image of western blot showing 24h and 48h of 0.9 µM TH1579 treatment on AXL and downstream effectors. **(D)** Densiometric analysis of western blot showing effect of TH1579 short term treatment (1 h, 3 h and 6 h) on AXL in A375VR4 and SkMel2 where for each set first bar is 1 h, second bar is 3 h and third bar is 6 h. **(E)** AXL and MTH1 mRNA expression after short-term exposure of CMM cells to TH1579 assessed by qPCR analysis.

**Figure S5: TH1579 reduces AXL expression in vivo but does not inhibit AXL in an *in vitro* kinase assay**

**(A)** Immunoflourescence shows that TH1579 at 20 µM is able to reduce total AXL expression for some tumor cells in both A375VR4 and SKMel2 xenograft zebrafish models. Arrows indicate tumor cells expressing AXL or tumor cells with loss of AXL expression. **(B)** *In vitro* kinase assay shows that TH1579 does not inhibit AXL activity. Here bemcentinib and carbozantinil, both known AXL inhibitors, have been used as positive controls.

**Figure S6: Biological processes regulated by TH1579 mediated molecular changes**

(A) Pathway analysis shows different Gene Ontologies regulated by TH1579 mediated changes. Pathway analysis was performed using NetworkAnalyst software (http://www.networkanalyst.ca/). **(B)** Melanoma comes up as topmost hit in the KEGG pathway analysis for TH1579 and the associated RTKs whose protein expression is affected after treatment.

**Figure S7: MTH1 and CAV-1 mRNA expression is correlated in CMM patients who respond to therapy**

**(A)** Alterations in CAV-1, AXL and MTH1 from TCGA (https://www.cancer.gov/tcga). TCGA analysis shows that these genes are altered in approximately 20% of CMM cases for AXL and MTH1 and in 25% for CAV-1 and MTH1 (n=479). **(B)** CAV1, AXL and MTH1 mRNA levels in tumors from CMM patients (classified here as responders or non-responders to targeted or immunotherapy). **(C)** Expression status of AXL, CAV-1 and MTH1 in CMM stratified on disease stage. mRNA and protein expression for the above mentioned genes based on TCGA data (n= 390, mRNA and n=191, protein). CAV-1 mRNA expression is significantly different in stage I-IV vs stage 0, whereas CAV-1 protein expression is different in stage II vs stage 0, although not reaching significance *p < 0.05, Mann-Whitney U test). **(D)** TCGA analysis (n=225, non-metastatic and n=195, metastatic) shows no correlation between metastatic or non-metastatic disease groups for AXL and MTH1 mRNA expression or MTH1 and CAV-1 mRNA expression. **(E)** No correlation is observed when comparing AXL and MTH1 (responders (n=25) and non-responders (n=7)), between CAV-1 and MTH1 (non-responders (n=7)). A positive correlation is observed between CAV-1 and AXL for non-responders to immunotherapy (n=4).

**Figure S8: Knockdown of CAV-1 decreases AXL and MTH1 expression in A375VR4 cells.**

**(A)** CAV-1 target engagement by TH1579 using CETSA shows no target engagement at 0.9 µM TH1579 treatment. CAV-1 signal is normalized to β-actin (loading control) and the maximal CAV-1 signal in the western blot. (The graph shows mean ± SEM; n = 3) **(B)** Representative western blot showing that silencing of CAV-1 also causes a decrease in MTH1 and AXL levels.

**Figure S9: CAV-1 interacts with MTH1**

**(A)** CAV-1 co-precipitates with MTH1 in a pull down assay. Co-IP performed on A375VR4 samples with CAV-1, MTH1 or IgG (negative control), followed by immunoblotting with CAV-1 antibody and MTH1 antibody. The results show a weak binding of CAV-1 to MTH1**. (B)** isPLA (In situ-proximity ligation assay) analysis was performed on A375, A375VR4 and SkMel2 using AXL and MTH1 antibodies. The results show that AXL does not interact with MTH1 in CMM cells as seen by no increase in the PLA signal count. **(C)** Quantification of (B).

**Figure S10: CMM cells show an additive effect when TH1579 is combined with BRAF inhibitors**

**(A)** Synergy plots showing synergy index with combination treatments. A375VR4 shows an synergy index ~2 when TH1579 is combined with vemurafenib. No synergy is observed when combining trametinib with TH1579 (n=3). **(B)** Combination treatment with TH1579 (0.2 µM) and vemurafenib (0.3 µM) reduces *BRAF* mutant cell line ESTDAB049 cell viability more than either drug alone. **(C)** Combination treatment of TH1579 with dabrafenib (a more widely clinically used BRAF inhibitor in *BRAF* mutant CMM cells is similarly able to reduce cell viability more effectively than the single drugs alone (error bars represent mean ± SD; n = 3; *p < 0.05, Student's t test). **(D)** Combining vemurafenib with TH1579 is able to significantly induce ROS levels when compared to either drug alone as seen by the increase in H2DCF-A counts (error bars represent mean ± SD; n = 3; *p < 0.05, **p<0.01, ***p<0.001, Student's t test).
