# Supplementary material for: AXL and CAV-1 play a role for MTH1 inhibitor TH1579 sensitivity in cutaneous malignant melanoma
Source: Cell Death Differ. 2020 Jan 9;27(7):2081–98. doi: 10.1038/s41418-019-0488-1 (PMC7308409; doi:10.1038/s41418-019-0488-1)
Supplement: Supplementary file 10 — Supplementary movie legends [file 41418_2019_488_MOESM10_ESM.docx]

**Supplementary movie MV1:** **Time lapse imaging showing A375 cells treated with DMSO**. A375 cells treated with DMSO have a mean time of 79 mins in the mitotic phase

**Supplementary movie MV2:** **Time lapse imaging showing A375 cells treated with 500nM TH1579**. A375 cells treated with 500nM TH1579 have significantly delayed mitosis with a mean time of 367 mins in the mitotic phase

**Supplementary movie MV3: Time lapse imaging showing A375VR4 cells treated with DMSO**. A375VR4 cells treated with DMSO have a mean time of 63 mins in the mitotic phase

**Supplementary movie MV4:** **Time lapse imaging showing A375VR4 cells treated with 500nM TH1579**. A375 cells treated with 500nM TH1579 have significantly delayed mitosis with a mean time of 444 mins in the mitotic phase

**Supplementary movie MV5:** **Time lapse imaging showing A375VR4 cells transfected with control shRNA**. A375VR4 cells transfected with scrambled control (shNT) have a mean time of 73 mins in the mitotic phase

**Supplementary movie MV6:** **Time lapse imaging showing A375VR4 cells transfected with shRNA against MTH1**. A375VR4 cells transfected with shMTH1 do not show any significant delay in mitotic phase with a mean time of 94 mins in the mitotic phase
